# Clinical Effects of *Streptococcus salivarius* K12 in Hospitalized COVID-19 Patients: Results of a Preliminary Study

**DOI:** 10.3390/microorganisms10101926

**Published:** 2022-09-28

**Authors:** Francesco Di Pierro, Somia Iqtadar, Sami Ullah Mumtaz, Alexander Bertuccioli, Martino Recchia, Nicola Zerbinati, Amjad Khan

**Affiliations:** 1 Scientific & Research Department, Velleja Research, 20100 Milan, Italy; 2 Digestive Endoscopy, Fondazione Poliambulanza, 25133 Brescia, Italy; 3 Department of Medicine, King Edward Medical University, Lahore 54000, Pakistan; 4 Department of Biomolecular Sciences (DISB), University of Urbino, 61029 Urbino, Italy; 5 Medistat, Unità di Epidemiologia Clinica e Biostatistica, 20100 Milan, Italy; 6 Department of Medicine and Surgery, University of Insubria, 21100 Varese, Italy; 7 Nuffield Division of Clinical and Laboratory Sciences (NDCLS), Radcliffe Department of Medicine, John Radcliffe Hospital, University of Oxford, Oxford OX3 9DU, UK

**Keywords:** oral microbiota, lantibiotics, salivaricins, *Streptococcus salivarius*, COVID-19, SARS-CoV-2

## Abstract

Anatomical and physiological considerations indicate that the oral cavity is a primary source of the lung microbiota community, and recent studies have shown that the microbiota in the lungs contributes to immunological homeostasis, potentially altering the organ’s susceptibility to viral infection, including SARS-CoV-2. It has been proposed that, in the case of viral infection, lung Gram-negative bacteria could promote the cytokine cascade with a better performance than a microbiota mainly constituted by Gram-positive bacteria. Recent observations also suggest that *Prevotella*-rich oral microbiotas would dominate the oral cavity of SARS-CoV-2-infected patients. In comparison, *Streptococcus*-rich microbiotas would dominate the oral cavity of healthy people. To verify if the modulation of the oral microbiota could have an impact on the current coronavirus disease, we administered for 14 days a well-recognized and oral-colonizing probiotic (*S. salivarius* K12) to hospitalized COVID-19 patients. The preliminary results of our randomized and controlled trial seem to prove the potential role of this oral strain in improving the course of the main markers of pathology, as well as its ability to apparently reduce the death rate from COVID-19. Although in a preliminary and only circumstantial way, our results seem to confirm the hypothesis of a direct involvement of the oral microbiota in the construction of a lung microbiota whose taxonomic structure could modulate the inflammatory processes generated at the pulmonary and systemic level by a viral infection.

## 1. Introduction

Recent clinical studies have suggested a possible direct relationship between the lung and the oral microbiotas [1]. Analysis of the bronchoalveolar lavage fluid (BALF) of both healthy subjects and COVID-19 patients has revealed the presence of elevated levels of oral and upper respiratory commensal bacteria [2]. Anatomical and physiological considerations indicate that the oral cavity is the primary source of the lung microbiota community, acquired via aspiration and inhalation [3,4]. The microbiota of the lungs overlaps in large extent with that found in the mouth. In humans, the prominent taxa in BALF samples include mainly *Streptococcus*, *Prevotella*, and *Veillonella*, and these have been detected in concurrently collected oral samples [3,5]. Recent studies have shown that the microbiota in the lungs contributes to immunological homeostasis and can potentially alter susceptibility to viral infection [6]. It has been proposed that the Gram-negative bacteria could promote an inflammatory cytokine cascade, with a better performance than a microbiota mainly constituted by Gram-positive bacteria [7]. It is possible that the lipopolysaccharide (LPS) released by Gram-negative bacteria could trigger a response constituted by inflammatory cytokines, such as TNF-α, IL-6, and IL-1, stronger than the one triggered by Gram-positive bacteria [8,9]. With respect to COVID-19, a particular abundance of *Prevotella* and *Veillonella* spp. in the lung and oral microbiotas’ composition has been observed in patients with SARS-CoV-2 pneumonia [10,11,12,13,14,15,16]. A recent study profiled the oral microbiota of healthy controls and COVID-19-hospitalized patients, discovering the existence of four different bacterial consortia, which the authors named Species Interacting Groups (SIGs) [17]. It is noteworthy that SIG1 and SIG4, respectively dominated mainly by *Prevotella* and *Veillonella* spp., were distinctive for COVID-19 pneumonia patients. Conversely, the same two taxa were not present or poorly represented in the SIGs distinctive of healthy controls, SIG2 and SIG3, which were instead characterized by the genus *Streptococcus*. Notably, the SIG2 consortium showed, among others, the presence of the species *S. salivarius*, an abundant representative of the normal oral consortium, also available as an oral probiotic [18]. Oral–pharyngeal bacteria are swallowed daily and can therefore potentially be detected, albeit at minimal concentrations, also in the faecal microbiota. Indeed, the *S. salivarius* species is poorly detected in the faecal consortium of subjects affected by COVID-19 [19]. The authors observed that SIG1 and SIG4, those characterizing the COVID-19 oral microbiota, correlated with the presence of IL-6, a pro-inflammatory cytokine involved in the well-known “cytokine storm” characterizing a severe COVID-19 condition, while SIG2 and SIG3, those characterizing the healthy control oral microbiota, did not [17]. The lung-protective role potentially expressed by the species *S. salivarius* should seem to be confirmed also in other lung pathologies, i.e., cystic fibrosis [20]. Taken altogether, these findings could suggest that some bacterial species, for instance those characterizing the beneficial SIGs, may be used as local probiotics to restore the oral microbiota as a public intervention during the pandemic [21]. The use of the strain *S. salivarius* K12 has been recently proposed as an oral probiotic treatment to reduce the risk of SARS-CoV-2 infection and has been demonstrated to reduce the rate of SARS-CoV-2 swab positivity, at least in children [22]. In a randomized and controlled clinical trial, aimed at evaluating the prophylactic role of oral probiotics in reducing bacterial and viral pharyngo-tonsillitis, 128 school-attending children within the Milan (Italy) area were enrolled and treated daily for 90 days (or received no treatment; control group) with *S. salivarius* K12. Due to symptoms, a nasal swab for the detection of the SARS-CoV-2-specific antigen was performed in 33 and in 46 children, in the treated and in the control groups, respectively. Positivity of the antigen swab was detected only in 24 children within the control group. No children in the group receiving probiotic *S. salivarius* K12 showed positivity in the test. Out of the 24 positive children, seven had parents with COVID-19, four had brothers and/or sisters testing positive by the swab test for SARS-CoV-2, and 13 had classmates testing positive for the swab test for SARS-CoV-2. This preliminary report supports the hypothesis that the oral administration of oral-colonizing bacteria belonging to SIG2 or SIG3 (*Streptococcus*, a Gram-positive bacteria) could afford protection from SARS-CoV-2 infection and/or severe COVID-19 disease development. It also supports the idea that the administration of orally colonizing bacteria belonging to the microbial consortia most frequently found in subjects not affected by COVID-19 (*Streptococcus*) is protective against infection. In a multicentre, randomized, and controlled clinical trial conducted in Wuhan (China), involving 200 front-line medical healthcare workers directly engaged in the treatment of COVID-19 patients, the use of *S. salivarius* K12 oropharyngeal probiotic dietary supplementation as a prophylactic significantly reduced the incidence of respiratory tract infections by 64.8% [23]. Based on its widely demonstrated efficacy and safety profile [24,25,26,27,28], we investigated the oral administration of *S. salivarius* K12 to hospitalized COVID-19 patients (not already in intensive care units (ICUs)) receiving supplementary oxygen (non-invasive oxygen therapy) to exploit the “ventilation” and helping *S. salivarius* (K12) move from the mouth (it is an “oral” bacteria) to the lungs, colonizing them. The idea is that the presence of *S. salivarius* K12 in the lungs could strategically reduce the lungs’ and immune capability to release pro-inflammatory cytokines, thus preventing excessive lung inflammation and the need to proceed to the ICU and death. The aim of our study was therefore to verify the safety and the possible efficacy profile of *S. salivarius* K12 on patients hospitalized because of COVID-19.

## 2. Materials and Methods

### 2.1. Study and Criteria

**Figure 1:**
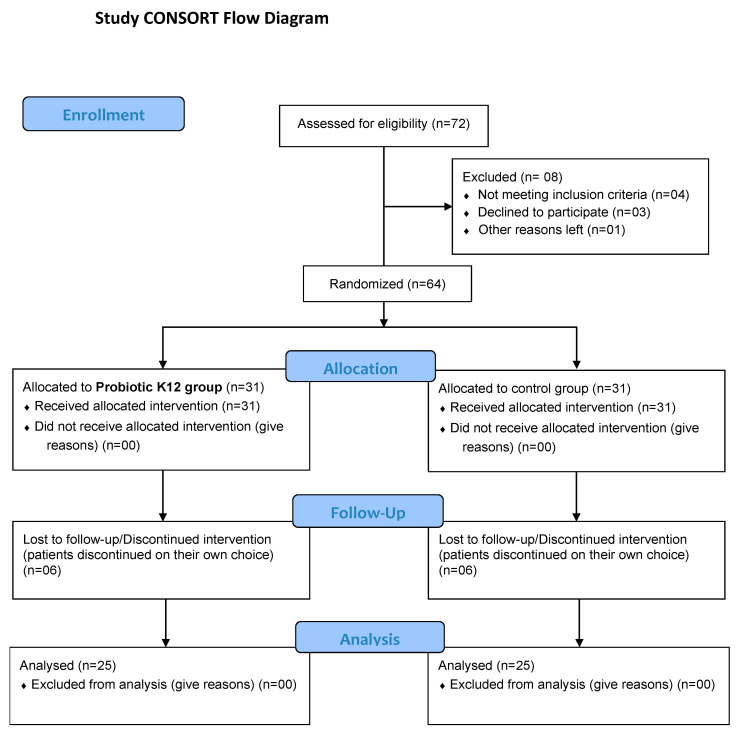
Protocol scheme adopted for the study.

This is a randomized, open-labelled, two-arm, single-centre, pilot clinical trial conducted at the Department of Medicine, King Edward Medical University (KEMU), Lahore, Pakistan. The study compared the treatment benefits of oral probiotic *S. salivarius* K12 plus standard of care (SOC) versus SOC alone in 50 hospitalized patients (non-ICU and not already receiving mechanical ventilatory support) with COVID-19 admitted to Mayo Hospital Lahore (a tertiary care 3000-bed teaching hospital) from 11 August 2021 to 18 November 2021. The study was approved by the Institutional Review Board (IRB) of King Edward Medical University, Lahore, via Ref. No. 625/RC/KEMU/07.09.2021 and has been registered on clinicaltrials.gov (accessed on 26 August 2022) with registration number NCT05043376. The inclusion criteria were: both sexes; above 18 years of age; confirmed SARS-CoV-2 infection shown by reverse-transcriptase-polymerase-chain-reaction (RT-PCR)-based positive nasopharyngeal swab; with typical acute COVID-19 symptoms including fever, cough, dyspnoea, pulmonary infiltrates on X-ray/CT, elevated inflammatory markers including C-reactive protein (CRP), D-dimers, lactate dehydrogenase (LDH), Ferritin; admitted to the hospital in the previous 48 h for the treatment of COVID-19 disease. The exclusion criteria were patients already in the ICU or those with the need for invasive mechanical ventilatory oxygen support at the time of hospital admission; patients with a history of hypersensitivity or allergic reaction to probiotics and any other condition or factor that, in the opinion of the treating consultant, contraindicates the use of a probiotic strain or makes the subject at risk due to his/her participation in the study. Informed written consent was obtained from the patients/the patient’s accompanying family member or relative before enrolling in the study. The study followed the Consolidated Standards of Reporting Trials (CONSORT) reporting guideline, and the flowchart of the study is shown in Figure 1.

### 2.2. Treatment Protocol

Hospitalized patients were randomly assigned (by the Block Randomization Algorithm) in a 1:1 ratio to receive either the standard of care (SOC) (control group) or SOC plus *S. salivarius* K12 (K12 group). The probiotic treatment scheme included slowly sucking two oral tablets per day for up to 14 days and was established to occur at night before sleeping, without biting or swallowing the tablets, but letting them slowly dissolve inside the mouth. The *S. salivarius* K12 treatment was stopped if the patient was safely discharge from the hospital before the 14th day, or if the patient remained admitted after the 14th day from enrolment, or if he/she was transferred to the ICU, or death. All patients were followed for up to 14 days for the clinical outcome defined as live discharge, or ICU transfer, or death or remaining admitted. Both K12 and control group patients received the same SOC treatment as per the hospital guidelines. These included corticosteroids, anticoagulants, antivirals, antibiotics, and proton pump inhibitors (PPIs). Clinical parameters and serum levels of inflammatory markers including CRP, D-dimers, LDH, and Ferritin were recorded at baseline and every two days during the 14 days of treatment. Pulmonary infiltrates on chest X-ray were evaluated only at enrolment.

### 2.3. Tested Product

The finished form of probiotic *S. salivarius* K12 used in the study (Bactoblis^®^; kindly and freely provided by Pharmextracta S.p.A., Pontenure, Italy) was registered as a food supplement at the Italian Minister of Health on 5 July 2011 (Registration Number 178/2002). Each oral-dissolving tablet contained more than 1 × 10^9^ colony forming units (CFU) of *S. salivarius* K12 (deposit number: ATCC BAA-1024).

### 2.4. Outcomes

Outcomes were improvement of biochemical parameters (CRP, D-dimer, Ferritin, LDH), fever, oxygen saturation level, need and length of oxygen therapy, the rate of progression to ICU and death.

### 2.5. Statistical Analysis

The study of the demographic data and the evaluation of the COVID-19 parameters were carried out using descriptive tables and graphs such as mosaic bar charts and box-and-whisker plots, which provide, with their dynamic function, a powerful tool for descriptive analysis. The evaluation of the parameters’ effectiveness (survival) and the trends of many laboratory indicators, such as CPR, D-dimer, LDH, Ferritin, and O_2_ saturation level, was conducted using parametric and non-parametric methods. We employed the Chiˆ2 test to capture information on efficacy between two different observation periods. We used the Cochran–Mantel–Haenszel test to assess relative risk in possible stratifications. We used the multiple logistic function to capture the contribution of certain predictors on the performance of laboratory indicators. All statistical evaluations used two-tailed tests and considered probability values < 0.05 as the significance level. Values between 0.05 and 0.10 described situations at the borderline of significance. The statistical software JMP14 (version 14.3.0) Pro of SAS Institute Inc. (Cary, NC, USA) was used for the analysis.

## 3. Results

Out of 50 hospitalized and enrolled patients, 47 completed the 14 days of treatment. Two were discharged upon request before the 14th day, one per group; one, belonging to the probiotic group, left the hospital against medical advice. The two discharged patients explain why in Figure 1 are described 64 patients randomised, but only 62 allocated. All patients were anyway considered valid for the analysis. The total sample of patients (N = 50) showed a mean age of 48.5 ± 15.4 years (45.5 ± 16.9 for the 21 females and 50.7 ± 14.1 for the 29 males). Concerning the age distribution, the whole sample of enrolled patients showed two main modal classes, corresponding to 30–40 and 50–60 years, having 22% of the total cases each, while patients corresponding to 20–30, 40–50, 60–70, and 70–80 years, respectively, represented 12%, 16%, 16%, and 12% of the total cases.

At enrolment, no significant differences were observed in the two groups of treatment for age and sex (Table 1), comorbidities (Table 2), blood biomarkers and saturimetry (Table 3), fever (data not shown; *p* = 0.0784), and shortness of breath (data not shown; *p* = 0.5389). As regards pulmonary infiltrates, we observed in both groups the same proportion of patients with normal, unilateral, or bilateral findings (data not shown; *p* = 0.9327). A slight difference was observed only for the need for supplementary oxygen, requested at Day 1 by 25 patients of the SOC group and by 21 of the K12 group (Table 4).

At enrolment, the pharmacological approach to patients completely overlapped between the two groups with no significant differences in terms of type, time, and dosage of drugs administration (data not shown).

To better understand the evolution of the different biomarkers during the 14 days of treatment, we considered their value at baseline (T1) and the last available value (Ti) for each patient. Then, for both groups, we counted the frequency of patients with a final value (Ti) lower or higher than the one observed at baseline (T1). This method allowed us to have a simple perspective of the proportion of patients improving or worsening during the trial according to the treatment group.

**Figure 2:**
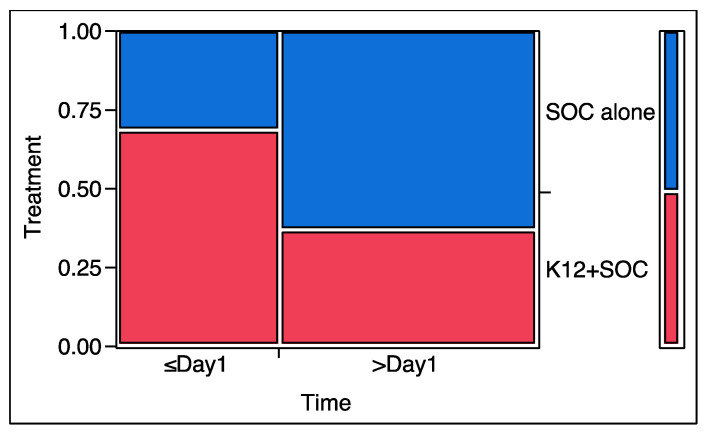
Patient proportion that demonstrates improvement (≤Day 1) or worsening (≥Day 1) of Ferritin according to the possible treatment (in blue, standard of care; in red, standard of care plus *S. salivarius* K12).

**Figure 3:**
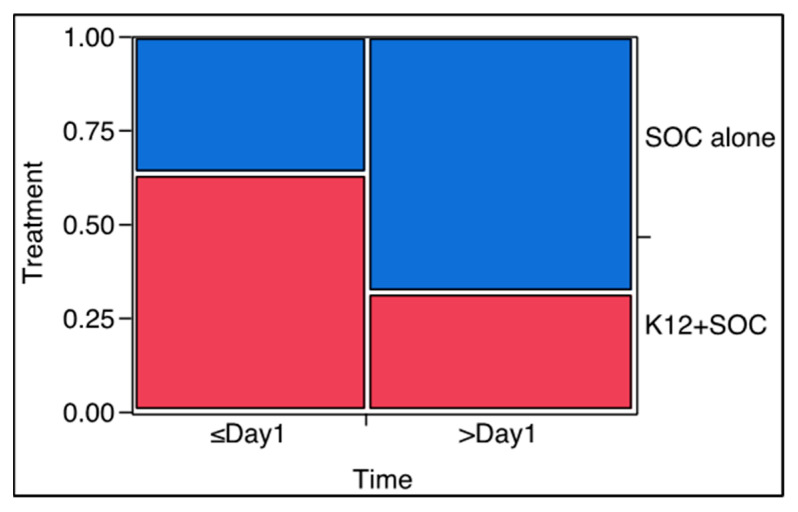
Patient proportion that demonstrates improvement (≤Day 1) or worsening (≥Day 1) of supplementary oxygen need according to the possible treatment (in blue, standard of care; in red, standard of care plus *S. salivarius* K12).

As shown in Table 5, in comparison with the control group, all parameters (CRP, D-dimer, LDH, Ferritin, oxygen saturation, fever, and supplementary oxygen) showed a better improvement in the K12 group, with significant results for Ferritin (*p* = 0.0303) and supplementary oxygen requirement (*p* = 0.0301). To better visualize the possible effect exerted by the administration of the oral probiotic versus the standard of care, the mosaic plots concerning Ferritin and supplementary oxygen need are shown in Figure 2 and Figure 3.

When entering the two parameters separately in a multiple logistic model with sensible regressors (“Sex”, “Hospitalisation Days”, “Age” (in years), and “Comorbidity”), the probability of having a lower Ferritin and supplementary oxygen values was for both parameters five-times higher for the K12 group versus the SOC group with *p* = 0.0264 and *p* = 0.0249, respectively (data not shown). Table 6 shows the results about the need to proceed to the intensive care unit (ICU) and the number of deaths. In the SOC group, 8 patients were transferred to the ICU before the end of the 14-day treatment period and 2 others died before being transferred to the ICU. Of the 8 patients promptly transferred to the ICU, 6 died within a few days. In the K12 group, 8 patients were promptly transferred to the ICU before the end of the 14 days of treatment. Of these, three died. Overall, mortality affected 32% of the patients in the SOC group versus 12% of the patients in the K12 group (odds ratio: 3.45098, CI95% 0.793374–15.0109), which means that survival was more than three-times higher in patients treated daily with the *S. salivarius* K12 strain than in patients not treated with the oral probiotic. To investigate the possible activity of confounding variables (CRP, D-dimer, LDH, Ferritin, and oxygen requirement), whose role could have affected this result, we used the Mantel–Haenszel test. As expected, CRP, D-dimer, LDH, Ferritin, and oxygen need resulted in being significantly correlated (data not shown) with the mortality rate (the higher the value, the higher the risk of death). However, this occurred regardless of the treatment protocol for CRP, D-dimer, LDH, and Ferritin. In contrast, the higher oxygen demand of the patients in the SOC group resulted in being significantly different (*p* = 0.0022) from that ascertained for the *S. salivarius*-K12-treated patients (Table 7).

## 4. Discussion

According to an accredited view among microbiota scholars, lung tissue, even in physiological conditions, would not be sterile [29], but on the contrary, it would contain a bacterial consortium largely inherited from the oral cavity through the processes of ventilation and micro-aspiration [30]. These daily processes would connect the two anatomical sites: mouth and lungs [31]. According to a further vision, the bacterial component physiologically present in the lungs could influence the inflammatory reactivity of the lung tissue itself, mainly in relation to the Gram-negative bacterial fraction [32]. The latter is in fact equipped with LPS, a known mediator of inflammatory processes mainly developed by macrophage release of TNF-α, IL-1β, and IL-6. Indeed, the lung bacterial consortium contains an important fraction of Gram-negative bacteria including Proteobacteria (mainly *Acinetobacter*, *Comamonas*, *Pseudomonas*, and *Ralstonia*), Bacteroidetes (mainly *Prevotella*), and Negativicutes (mainly *Veillonella*). It, obviously, also contains Gram-positive bacteria such as *Streptococcus* and *Staphylococcus* (both Firmicutes) [33]. In consideration of a different inflammatory potential (the LPS of bacteria from the phylum Proteobacteria is in fact hundreds of times more inflammatory than that of the bacteria belonging to the phylum Bacteroidetes and to the class of Negativicutes) [34], a good presence of Bacteroidetes to the total detriment of the Proteobacteria fraction [9] or, in an even more impactful way, a greater presence of Gram-positive bacteria (such as *Staphylococcus*) on the total pulmonary consortium could reduce the inflammatory background present in the lungs under physiological conditions [6]. According to a rather recent point of view, a more “primed” inflammatory background would favour, in the case of lung viral infection, a more consistent and more burdensome inflammatory cascade for the host than that which would develop with a less consolidated inflammatory background [8]. Even if it is not yet possible to speak of certain evidence, but only of strongly circumstantial aspects, these elements, in addition to what it was already explained in the “Introduction” Section, prompted us to verify the possibility of influencing the lung inflammatory background using an oral probiotic strain (*S. salivarius* K12).

Strain K12 has been widely clinically investigated especially for its effective action in contrasting ear, oral, pharyngeal, and tonsillar infections caused by *S. pyogenes*, *S. pneumoniae*, *M. catarrhalis*, and/or *H. influenzae* [35,36,37,38,39,40,41]. The effectiveness of the strain K12 is more often traced back to its ability to release two lantibiotics (Salivaricin A2 and Salivaricin B), effective at damaging the membrane of the target bacteria [42]. However, its counteracting capacity towards antagonists is not extinguished in the release of bacteriocins. Other microbial species, potentially linked with the oral habitat such as oral–pharyngeal viruses (syncytial virus, adenovirus, rhinovirus), fungi such as *Candida*, or Gram-negative bacteria such *Aggregatibacter*, *Fusobacterium*, or *Porphyromonas*, are effectively contrasted by the *S. salivarius* K12 strain within the oral–pharyngeal environment, surely not through the two salivaricins, which are considered ineffective [43,44,45].

The results obtained in our pilot trial seem to support our hypothesis. In fact, the use of the *S. salivarius* K12 strain, administered in a way to favour the oropharyngeal colonization, seemed to improve the clinical aspects of symptomatic and hospitalized patients with a diagnosis of COVID-19. The 14-day treatment with the *S. salivarius* K12 strain in fact improved the course of all the blood markers typically used to follow the inflammatory phenomena that characterize the COVID-19 patient and reduced the need to resort to supplementary-oxygen-based therapies. Although highly significant values were reached only for parameters such as Ferritin and oxygen demand, overall, our study highlighted the potential of a possible therapeutic approach based on the use of an oral probiotic, which, ultimately, seems to have reduced by about three-times the risk of patient’s death. Obviously, we have no elements of certainty that can mechanistically explain our, however preliminary, results.

Anyway, a first hypotheses brings us back to what has been “learned” about what happened in the past with SARS-CoV-1. In fact, it was observed that a possible discriminating element, capable of influencing the survival of patients with SARS-CoV-1 infection, was the patient’s ability to produce a prompt interferon response [46]. The use of the *S. salivarius* K12 strain has been shown to increase the host’s ability to produce a prompt interferon response not accompanied by a parallel inflammatory response based on TNF-α, IL-1β, and IL-6 [18]. While the ability to produce an interferon response in the host would seem horizontally shared by other *S. salivarius* strains [47], the inability to determine the concomitant presence of an inflammatory response would instead seem to concern the *S. salivarius* K12 strain in a peculiar way [48]. A prompt and rapid interferon response, notoriously anti-viral, could, at least theoretically, explain the observations made in our pilot study.

A second hypothesis could concern another cytokine, IL-12, endowed with a strong anti-viral action. The release of IL-12, exactly like the presence of *Streptococcus*, characterizes the oral habitat of COVID-19-free patients [17]. Again, the *S. salivarius* strains increase the IL-12 response in the colonized patient [47]. Obviously, nothing denies that this second hypothesis could go hand in hand with the first. It is therefore likely that the colonization processes with *S. salivarius*, first oral and then pulmonary, could determine the double increase of both interferon and IL-12, strongly impacting the anti-viral response of the host.

There is also a third hypothesis. Recently, it has been observed, in double-blind conditions and against placebo, that even the simple swallowing of high doses of *S. salivarius* K12, therefore with poor or no oral colonization, can enforce the patients’ response to viruses (it increases in the presence of plasma IL-12), generating at the same time an anti-inflammatory effect (by reducing the plasma IL-6 values) [49]. It is undeniable that also the patients of our study, apart from being or not potentially orally colonised, swallowed their own saliva every day. This can also contain *S. salivarius* strains in large quantities because of the therapeutic approach. It is therefore possible that treatment with the oral probiotic may have influenced the systemic anti-viral and anti-inflammatory responses, improving them, of the hospitalized patient.

Obviously, it is also possible that, more simply, *S. salivarius* K12, after colonizing the oropharyngeal environment, effectively antagonized the presence of Gram-negative oral species (*Prevotella*, *Veillonella*, *Fusobacterium*, etc.) and then moved itself into the lung environment, producing, at the same time, a minor oral–pulmonary migration of the Gram-negative species.

A possible interpretation of the results concerning the trends of Ferritin and oxygen demand during the trial (Figure 2 and Figure 3) and their relationship with the mortality rate and treatment allow us to consider a possible eubiotic impact of *S. salivarius* K12 on lung inflammatory status. If, on the one hand, we must admit that the significant difference observed between the two groups as regards Ferritin could be just a simple consequence of the patient’s better clinical condition and not the demonstration of a possible direct effect on immunity or on metabolic aspects commonly described to be affected in coronavirus-infected patients [50], on the other hand, the results obtained by analysing the oxygen demand and the death rate prompted us to propose the existence of a possible “therapeutic connection” between the oral and lung microbiota. This connection could have allowed *S. salivarius* K12 to enhance the chance of surviving COVID-19. In fact, the higher oxygen demand of the patients during the study in the SOC group resulted in being highly significantly different (*p* = 0.0022) from that ascertained during the study for the patients of the K12 group, demonstrating a possible direct role played by the oral probiotic on lung inflammation and functionality. In fact, the “raw” (SOC and SOC + K12) proportion of the total deaths with increasing oxygen demand was 42% (10/24). After stratifying the analysis by treatment, the proportion of deaths with increasing demand of oxygen in the SOC group corresponded to 47% (8/17) and the one for K12 to 29% (2/7). The first overlaps with the raw one (47% vs. 42%), while the second is surely different (29% vs. 42%). This prompted us to assume that the highest mortality could be linked to the treatment approach not involving *S. salivarius* K12.

Obviously, we must admit that we have no objective elements that can tell us what really happened. In fact: (1) we do not know the structure of the oral and pulmonary microbiota of the enrolled patients; (2) we do not have the data of oropharyngeal and pulmonary colonization following treatment with the oral probiotic; (3) we do not have the cytokine analyses that can show us the effects of the probiotic strain on interferons, IL-12, TNF-α, IL-1β, and IL-6. All these elements are indeed important and not the only limitations of our study. Not having used a placebo, not having worked in double-blind conditions, not having worked looking for a possible dose–response effect of *S. salivarius* K12, and having enrolled a surely small number of inpatients certainly also limit the validity of our results. While recognizing these limitations, our study has the advantage of being, to our knowledge at least, the first study ever performed that tried to highlight the impact of a probiotic strain, with proven oral colonization dynamics and for which it is possible to hypothesize the ability to colonize the lung tissue, on the clinical course of symptomatic and hospitalized patients affected by COVID-19.

As already said, the preliminary nature of our results obviously requires the carrying out of further clinical studies, double-blind and against placebo, which could confirm or refute what we have apparently observed. Of course, it will also be mandatory that subsequent studies are able to highlight the structure of the oral and pulmonary microbiotas of patients, before and after treatment, also showing clinicians the cytokine asset before and after the treatment with the oral strain.

## 5. Conclusions

In a randomized and controlled study, the adjuvant use of *S. salivarius* K12, an oral probiotic endowed with a well-known capability to colonize the oral environment, improved the blood parameters and reduced the death rate in COVID-19 patients.

## Figures and Tables

**Table 1:** Enrolled patients according to age, sex, and treatment.

	*Group SOC*	*Group K12*	*p = 0.2121*
N	25	25	
Sex: male/female	16/9	13/12	
Age (both sex)	51.3 ± 16.0	45.8 ± 14.6	
Male age	51.2 ± 15.4	50.2 ± 13.0	
Female age	51.4 ± 18.1	41.1 ± 15.3	

SOC: standard of care; K12: oral probiotic (+SOC).

**Table 2:** Comorbidities of the enrolled patients.

*Pathologies*	*Number of Patients*		
	*Group SOC*	*Group K12*	*p = 0.5436*
Allergic asthma	1	0	
Autoimmune disorder (AD)	1	1	
Non-Hodgkin’s lymphoma	0	1	
Chronic kidney disease (CKD)	0	1	
Diabetes mellitus (DM)	1	1	
CKD + DM	1	1	
Chronic liver disease	2	0	
Coronary artery disease (CAD)	2	0	
Dengue fever	0	1	
DM + AD	0	1	
DM + breast cancer	1	0	
DM + hypercholesterolemia	0	2	
Hypertension (Ht)	2	7	
DM + CAD + Ht	2	1	
DM + obesity	0	2	
DM + psoriasis	0	1	
DM + Ht	2	0	
DM + Ht + others	0	1	
DM + others	0	1	
Others	10	3	
Mean	1.72	1.68	

SOC: standard of care; K12: oral probiotic (+SOC); others: functional gastrointestinal diseases, acne, rosacea, vaginal, bladder, or prostate discomfort.

**Table 3:** Blood parameters and percentage of O_2_ saturation in the enrolled patients.

*Parameters (M ± SD)*	*Group SOC*	*Group K12*	p *
CRP	100.28 ± 56.71	75.60 ± 41.95	0.0872
D-dimer	1.99 ± 1.16	2.33 ± 1.76	0.4146
LDH	460.24 ± 186.61	400.68 ± 175.97	0.2514
Ferritin	910.44 ± 528.57	793.58 ± 467.46	0.4161
O_2_ saturation (%)	93.72 ± 1.40	92.58 ± 2–95	0.0963

SOC: standard of care; K12: oral probiotic (+SOC); M ± SD: mean ± standard deviation. *: *t*-test assuming unequal variances.

**Table 4:** Number of patients with need for supplementary oxygen at enrolment.

*Volume O_2_ (L/min)*	*SOC (%)*	*K12 (%)*	*p = 0.0416*
0	2 (8)	5 (23.81)	
1	0 (0)	1 (4.76)	
2	0 (0)	4 (19.05)	
3	2 (8)	0 (0)	
4	1 (4)	0 (0)	
5	2 (8)	5 (23.81)	
8	4 (16)	2 (9.52)	
10	4 (16)	0 (0)	
12	4 (16)	2 (9.52)	
15	3 (12)	2 (9.52)	
20	3 (12)	0 (0)	
Total patients	25 (100)	21 (80.95)	

SOC: standard of care; K12: oral probiotic (+SOC); (L/min): litres per minute.

**Table 5:** Number of patients with a value ≤ or > than the one observed at enrolment.

*Parameter*	*SOC*		*K12*		p *
	*≤Day 1*	*>Day 1*	*≤Day 1*	*>Day 1*	
CRP	14	11	16	9	0.5637
D-dimer	12	13	14	11	0.5713
LDH	9	16	15	10	0.1573
Ferritin	6	19	13	11	0.0303
Oxygen saturation	7	18	6	18	0.8121
Fever	23	2	25	0	0.1489
Supplementary oxygen	8	17	14	8	0.0301

SOC: standard of care; K12: oral probiotic (+SOC); (L/min): litres per minutes. *: Pearson Chiˆ2 test.

**Table 6:** Number of patients transferred to the ICU, recovered, or deaths.

	*SOC (%)*	*K12 (%)*
Died before being transferred to ICU	2	0
Transferred to ICU	8	8
Died in ICU	6	3
Recovery from ICU	2	5
Total deaths	8	3

ICU: intensive care unit; SOC: standard of care; K12: oral probiotic (+SOC).

**Table 7:** Relationship between death and lower or higher supplementary oxygen request according to the treatment group.

*Parameter*	*SOC*		*K12*		* p = 0.0022
	***Lower***	***Higher***	***Lower***	***Higher***	
Dead	0	8	0	2	
Alive	8	9	15	5	

SOC: standard of care; K12: oral probiotic (+SOC); lower: patients whose request for oxygen during the study was reduced versus the oxygen request at enrolment; higher: patients whose request for oxygen was increased versus oxygen requested at enrolment. The total number of patients of the SOC group is 25. The total number of patients of the K12 group is 22 (3 patients did not need supplementary oxygen). *: Cochran–Mantel–Haenszel test.

## Data Availability

Data related to this manuscript can be made available from the corresponding author upon reasonable request.
